# IAP-TransUNet: integration of the attention mechanism and pyramid pooling for medical image segmentation

**DOI:** 10.3389/fnbot.2025.1706626

**Published:** 2025-12-01

**Authors:** Yuxuan Shi, Fang Li, Shuting Zhao, Hongmeng Yu, Xinrong Chen, Quan Liu

**Affiliations:** 1 ENT Institute and Department of Otolaryngology, Eye & ENT Hospital of Fudan University, Shanghai, China; 2 School of Computer Science and Technology, Shanghai University of Electric Power, Shanghai, China; 3 College of Biomedical Engineering, Fudan University, Shanghai, China

**Keywords:** transformer, attention mechanism, pyramid pooling, medical image segmentation, lightweight network

## Abstract

**Introduction:**

The combination of CNN and Transformer has attracted much attention for medical image segmentation due to its superior performance at present. However, the segmentation performance is affected by limitations such as the local receptive field and static weights of CNN convolution operations, as well as insufficient information exchange between Transformer local regions.

**Methods:**

To address these issues, an integrated attention mechanism and pyramid pooling network is proposed in this paper. Firstly, an efficient channel attention mechanism is embedded into CNN to extract more comprehensive image features. Then, CBAM_ASPP module is introduced into the bottleneck layer to obtain multi-scale context information. Finally, in order to address the limitations of traditional convolution, depthwise separable convolution is used to achieve a lightweight network.

**Results:**

The experiments based on the Synapse multi organ segmentation dataset and ACDC dataset showed that the proposed IAP-TransUNet achieved Dice similarity coefficients (DSCs) of 78.85% and 90.46%, respectively. Compared with the state-of-the-art method, for the Synapse multi organ segmentation dataset, the Hausdorff distance was reduced by 2.92%. For the ACDC dataset, the segmentation accuracy of the left ventricle, myocardium, and right ventricle was improved by 0.14%, 1.89%, and 0.23%, respectively.

**Discussion:**

The experimental results demonstrate that the proposed network has improved the effectiveness and shows strong performance on both CT and MRI data, which suggests its potential for generalization across different medical imaging modalities.

## 1 Introduction

Medical image segmentation aims to locate and segment lesion areas or organ tissues in medical images to help doctors make accurate and efficient diagnoses (Kepp et al., 2025). It is a crucial step in medical image analysis. Medical images, as a special type of image, have more blurred edges, lower contrast, and more complex shapes, which present some difficulties and challenges for segmentation tasks (Gülmez, 2025).

With the widespread application of deep learning in medical image processing, automatic feature learning algorithms based on deep learning have become an appropriate method for medical image segmentation, resulting in the rapid development of medical image segmentation. For example, deep learning models, such as convolutional neural networks (CNNs), are characterized by high speed, high accuracy, and automation (Sarvamangala and Kulkarni, 2022; Anderson et al., 2025; Yu et al., 2021). Their success is evident not only in image analysis tasks but also in broader applications such as predicting protein function and toxicity (Le, 2019; Zhao et al., 2022). Based on traditional CNNs and the encoding–decoding structure, a fully convolutional network (FCN) (Long et al., 2015) was proposed to capture position information by changing the fully connected layer to a convolution layer. In the FCN, transposed convolution is used for up-sampling to obtain segmented images with rich semantics. Subsequently, the U-Net network was proposed based on the FCN (Ronneberger et al., 2015), introducing a U-shaped network structure that was used for the first time for medical image segmentation. In the U-Net network, the encoding process extracts semantic information, the decoding process restores spatial dimensions, and skip connections are introduced to connect feature information at the same level. Furthermore, with the improvement of U-Net, a series of variants emerged. Among them, the Res-UNet network (Jiang et al., 2024) simplifies the training of the network by introducing multiple residual blocks in the encoder and the decoder, effectively alleviating the problems of gradient vanishing and semantic loss. A dense connection structure is introduced in the DenseUNet network (Guan et al., 2019) to avoid overfitting problems, enhance the transmission and reuse of the features, and significantly reduce the number of network parameters. To improve the ability to extract and fuse features, the U-Net++network (Zhou et al., 2019) reduces semantic differences between encoder and decoder feature maps by redesigning skip connections in the network. The UNet 3+ network (Huang et al., 2020) fuses feature maps of different scales through full-scale skip connections and achieves full-scale depth monitoring by calculating the loss between the fused feature maps and manually annotated data. Therefore, it can be seen that a U-shaped network combined with residual multi-scale feature fusion is beneficial for medical image segmentation (Pu et al., 2024).

With the application of CNNs, their inherent limitations have become apparent, including the limited receptive field of convolution operations, which can only perceive local feature information and cannot effectively capture global dependencies and interactions. With the success of the transformer (Vaswani et al., 2017) and its introduction into computer vision, the vision transformer (ViT) was proposed (Dosovitskiy et al., 2020). The model's outstanding performance on image classification tasks has demonstrated the great potential of the transformer in the field of computer vision. Then, the SETR model (Zheng et al., 2021), based on the transformer encoder, was proposed for medical image segmentation, which integrates semantic information into the transformer architecture; however, it still exhibits certain limitations in extracting local features. Therefore, combining CNNs with transformers can fully utilize their respective advantages. A representative work, TransUNet (Chen et al., 2021), integrates CNNs and transformers, combining the strengths of both. To extract global contextual information, labeled image patches from CNN feature maps are encoded into an input sequence. The encoded features then undergo up-sampling and are fused with high-resolution CNN feature maps. However, the CNN is used for feature extraction and up-sampling, resulting in an excessively small receptive field for convolutions in both the encoder and decoder. Therefore, there is still room for improvement when applying TransUNet to medical images of different modalities.

In this study, an improved medical image segmentation network, IAP-TransUNet, is proposed, which integrates an attention mechanism and pyramid pooling.

The main contributions can be summarized as follows:

(1) To significantly reduce model complexity and improve feature extraction, an efficient channel attention (ECA) mechanism is embedded in the CNN.
(2) A CBAM_ASPP module is introduced into the bottleneck layer to obtain multi-scale context information before up-sampling, so as to make more accurate predictions.
(3) Depthwise separable convolution, instead of conventional convolution, is used to achieve higher computing efficiency, resulting in a lightweight network.

## 2 Related work

### 2.1 Transformer

The transformer, based on a self-attention mechanism, is applied to machine translation tasks (Wolf et al., 2020). With subsequent improvements, it has been applied to computer vision tasks (Han et al., 2022), achieving favorable results. By transforming the input vector into three distinct matrices—the query matrix *Q*, the key value matrix *K*, and the value matrix *V*—the calculation of the self-attention mechanism is given in Equation 1.


$$\begin{array}{l} Attention\left(Q,K,V\right)=softmax\left(\frac{QK^{T}}{\sqrt{d_{k}}}\right)\times V \end{array}$$ (1)

where *QK*^*T*^ is the attention score, *d*_*k*_ is the dimension of the query matrix *Q* and key value matrix *K*, and $\sqrt{d_{k}}$ is the scale factor.

The multi-head self-attention (MSA) mechanism is a combination of multiple attention mechanisms, each of which is calculated first and then concentrated to obtain the final output, as shown in Equations 2, 3.


$$\begin{array}{l} head_{i}=Attention\left(QW_{i}^{Q},KW_{i}^{K},VW_{i}^{V}\right) \end{array}$$ (2)

$$\begin{array}{l} MSA\left(Q,K,V\right)=Concat\left(head_{1},head_{2},\cdots \;,head_{h}\right)W^{o} \end{array}$$ (3)

Where $W_{i}^{Q},W_{i}^{K},W_{i}^{V}$ represent the linear transformation matrices of *Q, K*, and *V* of the *i*-th self-attention mechanism, *h* is the number of self-attention mechanisms, and *W*^*O*^ represents the weight matrix of the multi-head attention.

The multilayer perceptron (MLP), shown in Equation 4, is a linear combination.


$$\begin{array}{l} MLP\left(X\right)=\max\left(0,XW_{1}+b_{1}\right)W_{2}+b_{2} \end{array}$$ (4)

Where *X* is the input vector and *W*_1_, *b*_1_ and *W*_2_, *b*_2_ represent the weight matrix and the bias vector of two fully connected layers, respectively.

Position encoding is a method used to express the position information of elements in the sequence data. Position encoding information can be input in parallel to significantly improve computing efficiency. The encoding method is given in Equations 5, 6.


$$\begin{array}{l} PE_{\left(pos,2i\right)}=\sin\left(pos/10000^{2i/d}\right) \end{array}$$ (5)

$$\begin{array}{l} PE_{\left(pos,2i+1\right)}=\cos\left(pos/10000^{2i/d}\right) \end{array}$$ (6)

Where *pos* represents the position of an element in the sequence, *d* is the dimension of position encoding, 2*i* is the even dimension, and 2*i* + 1 is the odd dimension.

### 2.2 TransUNet

In TransUNet, the transformer layer is integrated into U-Net, enabling the network to combine the advantages of both the transformer and U-Net, resulting in remarkable effectiveness in medical image segmentation. It not only overcomes the limitations of the CNN in handling remote dependencies but also compensates for the lack of detailed positioning of the transformer. TransUNet mainly consists of three parts: a hybrid encoder, a cascaded up-sampler, and a segmentation head.

The hybrid encoder is composed of a CNN and a transformer, and its role is to map the pixel space of the original image into a multi-level feature space. First, the original image is input into the CNN to extract high-level features and retain some intermediate- and low-level features for fusion with the up-sampled features. Then, the global relationships among the image pixels are obtained by feeding the high-level features into the transformer.

Multiple cascaded sampling blocks form a cascaded sampler, which is used for decoding high-level features. To prevent the loss of detailed information in the process of image restoration and ensure accuracy, the decoder up-samples the encoded high-level features and then concatenates them with the low-level features stored in the encoder to achieve accurate positioning.

The segmentation head is the part that produces the result of image segmentation and implements the prediction of the segmentation mask. The convolution of 3 × 3 is used to obtain the segmented image, then the cross-entropy loss and the Dice loss are, respectively, used to calculate the segmentation loss and the classification loss, which are weighted and averaged to obtain the final loss to realize image segmentation.

Beyond TransUNet, several other hybrid architectures have advanced the field of medical image segmentation. For instance, Swin-UNet (Cao et al., 2022) leverages the hierarchical structure and shifted window mechanism of the Swin Transformer within a U-shaped encoder–decoder framework. To enhance performance in complex scenarios, BiSeg-SAM (Su et al., 2024) integrates powerful zero-shot segmentation capability into a domain-specific framework. Similarly, works such as the SDPT (Cao et al., 2024) introduce novel transformer-based architectures to better capture both local and global features, thereby advancing segmentation accuracy. These methods highlight a trend toward hierarchical features and advanced attention, motivating the improvements made in IAP-TransUNet.

## 3 Methods

### 3.1 Architecture of the IAP-TransUNet network

**Figure 1:**
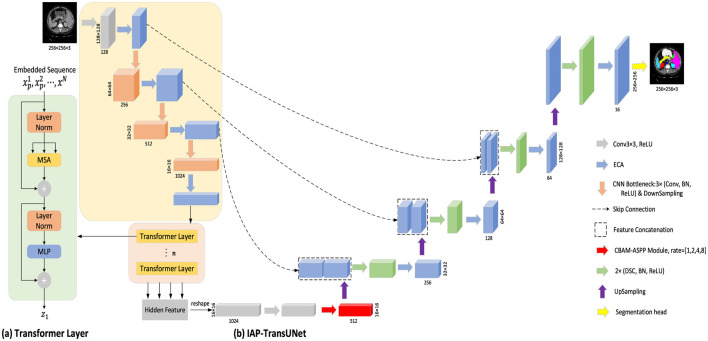
Overall framework of IAP-TransUNet. **(A)** Schematic diagram of the transformer layer; **(B)** The architecture of the proposed IAP-TransUNet.

TransUNet is a hybrid model that combines a CNN and a transformer to better handle global and local information. To fuse the high-resolution feature map in the encoder with the feature map in the decoder to obtain sufficient information, the feature map is first extracted using a CNN, then transformed and input into a transformer. Finally, the encoded feature map is up-sampled and fused with the high-resolution feature map in the encoder through skip connections. Based on TransUNet, this study proposes a new encoder–decoder model, IAP-TransUNet, by improving its network structure and integrating the ECA mechanism, the CBAM-ASPP module, and depthwise separable convolution, as shown in Figure 1. To use the proposed IAP-TransUNet model, an image is decomposed into patches of 256 × 256 × 3 before being sent to the input layer of the network, and after a series of processing in the network layers, the final pixel-level prediction is obtained in the last layer. TransUNet consists of **12** encoder layers, with an embedding dimension of **512**. Within each layer, the multi-head self-attention mechanism employs **12** attention heads, and the MLP has a hidden dimension of **2,048**.

### 3.2 Efficient channel attention (ECA) mechanism

**Figure 2:**
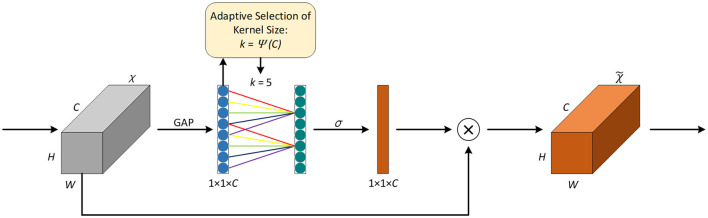
Attention mechanism of ECA.

Introducing attention mechanisms in neural networks can help focus on the key information of the input and invest computing power in the important areas to improve the efficiency and accuracy of the model. The squeeze-and-excitation network SENet (Hu et al., 2018) is a classic implementation of the channel attention mechanism; however, capturing all channels may reduce the efficiency of the model. It is known that convolution has a strong ability for information acquisition across channels. Wang et al. (2020) proposed the efficient channel attention network ECANet, which replaces the fully connected layer in the original SE module with one-dimensional convolution with a kernel size of *k* to avoid the dimension reduction of the channel and reduce the computation and complexity of the model while achieving higher accuracy. The network structure is shown in Figure 2.

First, the feature map χ from the previous layer is fed into the ECA module, χ∈*R*^*W*×*H*×*C*^, where *W, H*, and *C* represent the width, height, and number of channels, respectively. Then, global average pooling (GAP) is applied to χ to obtain the vector *g*(χ) of 1 × 1 × *C*, and the computation method of GAP is explained in Equation 7.


$$\begin{array}{l} g\left(\chi \right)=\frac{1}{W\times H}\sum_{I=1,J=1}^{W,H}\chi_{ij} \end{array}$$ (7)

Given the vector *g*(χ), information interaction across channels is performed according to Equation 8 to obtain the weight of each channel. The ECA module is implemented using a quick one-dimensional convolution with a kernel size of *k*, as shown in Equation 9.


$$\begin{array}{l} \omega_{i}=\sigma \left(\sum_{j=1}^{k}\omega^{j}y_{i}^{j}\right),y_{i}^{j}\in \Omega_{i}^{k} \end{array}$$ (8)

$$\begin{array}{l} \omega =\sigma \left(C1D_{k}\left(y\right)\right) \end{array}$$ (9)

Where ω_*i*_ is the weight of the *i*-th channel, σ is the sigmoid activation function, *y*_*i*_ represents the feature of the *i*-th channel, $y_{i}^{j}$ represents the feature of the *j*-th adjacent channel of the *i*-th channel, and $\Omega_{i}^{k}$ is the set of *k* adjacent channels. *C*1*D* represents one-dimensional convolution, and the size of the convolution kernel *k* is adaptively determined by Equation 10.


$$\begin{array}{l} k=\psi \left(C\right)={\left|\frac{\log_{2}\left(C\right)}{\gamma }+\frac{b}{\gamma }\right|}_{odd} \end{array}$$ (10)

Where γ = 2, *b*=1, and |*t*|_*odd*_ represents the odd number closest to *t*. In Figure 2, the value of *k* is 5.

Finally, by applying the weight obtained from Equation 9 to the input feature map, the resulting output feature map $\tilde{\chi }$ is obtained.

In IAP-TransUNet, ECA is integrated into the CNN part of the CNN–transformer hybrid encoder of the model. In addition, it is added after each convolution layer in the decoder path.

### 3.3 CBAM-ASPP module

**Figure 3:**
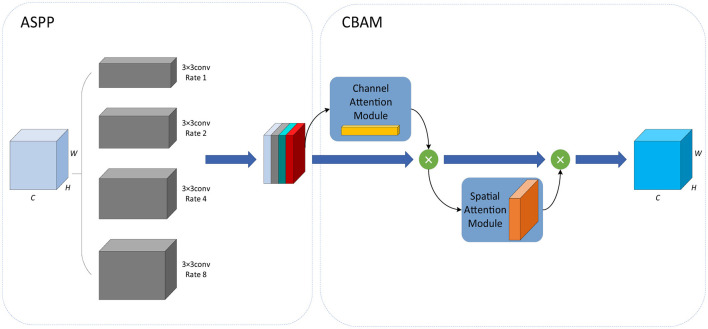
CBAM-ASPP module.

Based on the idea of spatial pyramid pooling (SPP) (He et al., 2015), the ASPP (Chen et al., 2017) module is the combination of SPP and dilated convolution. It employs multiple dilated convolutions with different sampling rates, which can capture local information of different scales so as to obtain the feature maps of different receptive fields. The Convolutional Block Attention Module (CBAM) (Woo et al., 2018) module introduces a spatial attention mechanism into a channel attention mechanism, consisting of the channel attention module (CAM) and spatial attention module (SAM). It can continuously generate attention feature maps of channels and spatial dimensions, and the final feature map is generated by multiplying them with the input feature map for adaptive feature correction. To further enhance the feature extraction capability of the ASPP module, Zhu et al. (2022) integrated the CBAM module into the ASPP module and proposed the CBAM-ASPP module, as shown in Figure 3. It makes the two modules complement each other to enhance the extraction of context information of different scales.

Firstly, in the ASPP module, 3 × 3 convolutions with four different dilation rates of 1, 2, 4, and 8 are used to extract features from the input data, which are then merged with the output.

Next, in the CBAM module, the output of the ASPP module is sequentially passed through the spatial attention module and the channel attention module.

In the channel attention module, by average pooling and max pooling, the average pooling feature *F*_*avg*_ and the max pooling feature *F*_max_ are obtained, each of which is then input into the same MLP, and the sigmoid function σ is used to obtain the channel attention coefficient *M*_*C*_, which is shown in Equation 11.


$$\begin{array}{l} M_{C}\left(F\right)=\sigma \left(MLP\left(F_{avg}\right)+MLP\left(F_{max}\right)\right) \end{array}$$ (11)

In the spatial attention module, average pooling and max pooling are applied to the output of the channel attention module to obtain the average pooling feature and the max pooling feature. Next, the two features are concatenated together according to the channel characteristics, then the convolution operation *f*^7 × 7^ with a kernel size of 7 × 7 and the sigmoid function σ are conducted successively to obtain the spatial attention coefficient *M*_*S*_. The calculation formula is shown in Equation 12.


$$\begin{array}{l} M_{s}\left(F\right)=\sigma \left(f^{7\times 7}\left(\left[AvgPool\left(F\right);MaxPool\left(F\right)\right]\right)\right) \end{array}$$ (12)

In our network, the CBAM-ASPP module is introduced into the bottleneck layer of IAP-TransUNet, aiming to obtain multi-scale contextual information before up-sampling.

### 3.4 Depthwise separable convolution

**Figure 4:**
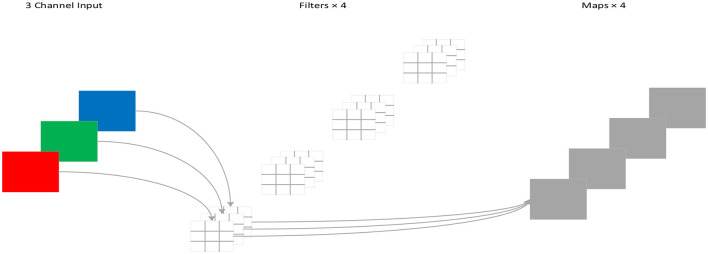
Standard convolution process.

**Figure 5:**
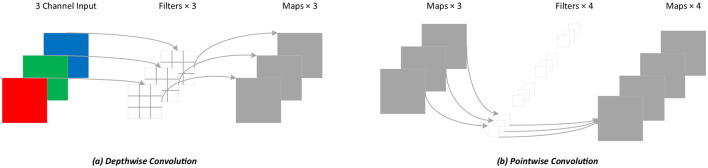
Depthwise separable convolution process. **(A)** Depthwise convolution; **(B)** Pointwise convolution.

For standard convolution, both spatial features and channel features are learned during the process of convolution, as shown in Figure 4. DSC, proposed by Howard et al. (Chollet, 2017), decouples the spatial correlation and channel correlation of the convolution layer into depthwise convolution and pointwise convolution through a transition layer, as shown in Figure 5. Compared to standard convolution, DSC considers the spatial correlation and channel correlation, respectively, greatly reducing the number of parameters and computation cost.

Assuming that the input and the output feature maps are the same and the convolution kernel used is *D*_*k*_×*D*_*k*_, the calculation of standard convolution is shown in Equation 13.


$$\begin{array}{l} D_{k}\times D_{k}\times M\times N\times D_{f}\times D_{f} \end{array}$$ (13)

Where *D*_*k*_ is the size of the convolution kernel *k, M* is the number of channels in the input feature map, *N* is the number of channels in the output feature map, and *D*_*f*_ is the width and height of the input and output feature maps *f*.

The calculation of DSC is shown in Equation 14, and pointwise convolution is implemented according to Equation 15. Therefore, the total calculation of DSC is shown in Equation 16.


$$\begin{array}{l} D_{k}\times D_{k}\times M\times D_{f}\times D_{f} \end{array}$$ (14)

$$\begin{array}{l} M\times N\times D_{f}\times D_{f} \end{array}$$ (15)

$$\begin{array}{l} D_{k}\times D_{k}\times M\times D_{f}\times D_{f}+M\times N\times D_{f}\times D_{f} \end{array}$$ (16)

The ratio of DSC to standard convolution can be obtained from Equations 13, 16, as shown in Equation 17.


$$\begin{array}{l} \frac{D_{k}\times D_{k}\times M\times D_{f}\times D_{f}+M\times N\times D_{f}\times D_{f}}{D_{k}\times D_{k}\times M\times N\times D_{f}\times D_{f}}=\frac{1}{N}+\frac{1}{D_{k}^{2}} \end{array}$$ (17)

From the equations above, DSC can significantly reduce the number of parameters and the computation cost. Since the proportion of computation is related to the kernel size *D*_*k*_ and the number of channels *N*, DSC achieves higher efficiency when *D*_*k*_ and *N* are larger.

In our network, the original convolution in the decoder of TransUNet is replaced with DSC to reduce the number of parameters and the computation cost.

## 4 Experimental results

### 4.1 Datasets

In total, two datasets were used: The Synapse multi-organ segmentation dataset (Synapse) and the Automated Cardiac Diagnosis Challenge (ACDC).

Synapse has 3,779 axial enhanced CT images, covering eight abdominal organs, including the aorta, gallbladder, left kidney, right kidney, liver, pancreas, spleen, and stomach, from 30 patients. In the dataset, 18 cases (2,212 axial slices) are used as the training set, and the remaining 12 cases (1,567 axial slices) are used as the test set.

ACDC is a cardiac MRI dataset consisting of 100 patients. For each case, the corresponding labels include the left ventricle, myocardium, and right ventricle. The dataset is divided into 70 training cases (1,304 axial slices), 10 validation cases (182 axial slices), and 20 test cases.

### 4.2 Experimental setup

The experiments were conducted in an environment with Python 3.7 and PyTorch 1.8.1, using an NVIDIA RTX 3090 GPU with 24 GB of memory. To increase data diversity and prevent overfitting, a standard on-the-fly data augmentation strategy was employed during training. This involved applying both horizontal and vertical flips, each with a probability of 0.5, and randomly rotating the images within an angle range of [−10, 10] degrees. This specific augmentation set was chosen to enhance model robustness while avoiding unrealistic anatomical distortions (Namozov and Im Cho, 2018). The detailed parameter settings were as follows: an input image resolution of 256 × 256, a patch size of 16, a batch size of 24, and expansion rates of the CBAM-ASPP module [1,2,4,8]. In addition, the model was trained using the SGD optimizer (Zhang et al., 2018) with a momentum of 0.9, a learning rate of 0.01, and a weight decay of 0.0001.

### 4.3 Loss function

For cases where the variation area in medical images is small, although the Dice loss is widely used, there may be a problem of gradient vanishing. Therefore, this study adopts a combination of the cross-entropy loss (Ho and Wookey, 2019) and Dice loss (Zhang et al., 2021).

The cross-entropy loss *L*_*CE*_ evaluates the difference between the model output and the true label and can measure the classification loss of pixels during the segmentation process. Its calculation is shown in Equation 18.


$$\begin{array}{l} L_{CE}=\frac{1}{N}\underset{i}{\sum }L_{i}=-\frac{1}{N}\underset{i}{\sum }\overset{M}{\underset{c=1}{\sum }}y_{ic}\log\left(p_{ic}\right) \end{array}$$ (18)

where *M* is the number of classes and *y*_*ic*_ refers to whether the true class of sample *i* is *c (1 if it is c, otherwise 0)*. *p*_*ic*_ refers to the prediction probability that sample *i* belongs to class *c*.

The similarity between the predicted segmented image and the ground truth image is evaluated using the Dice loss *L*_*Dice*_, with a value range of 0 to 1, as shown in Equation 19.


$$\begin{array}{l} L_{Dice}=1-\frac{2|X⋂Y|}{\left|X|+|Y\right|} \end{array}$$ (19)

where |*X*⋂*Y*| represents the intersection between the true image and the predicted image, while |*X*| and |*Y*| represent the number of elements, respectively.

The total loss function of the IAP-TransUNet model is shown in Equation 20.


$$\begin{array}{l} L=\frac{L_{CE}+L_{Dice}}{2} \end{array}$$ (20)

### 4.4 Evaluation indicator

This study used the Dice similarity coefficient (DSC) (Bertels et al., 2019) and Hausdorff Distance (HD) (Kreveld et al., 2022) to evaluate the performance of the model. The DSC imposes stronger constraints on the internal filling of segmented pixels, while the HD is more sensitive to segmentation boundaries. In addition, to evaluate the lightweight network of the model, the number of parameters, inference speed, and GFLOPS (Wang et al., 2022) were compared.

The DSC index is used to evaluate the similarity between the comparison targets, as shown in Equation 21. A larger value indicates greater similarity, indicating that the predicted result is closer to the benchmark,


$$\begin{array}{l} DSC\left(A,B\right)=\frac{2|A⋂B|}{\left|A|+|B\right|} \end{array}$$ (21)

where *A* is the true result and *B* is the predicted result.

The HD measures the distance between two point sets. It calculates the maximum value of the shortest distance between the predicted result and the true result, as shown in Equation 22.


$$\begin{array}{l} HD\left(A,B\right)=\max\left\{\underset{a\in A}{\max}\underset{b\in B}{\min}d\left(a,b\right),\underset{b\in B}{\max}\underset{a\in A}{\min}d\left(a,b\right)\right\} \end{array}$$ (22)

where *d*(*a, b*) is the Euclidean distance between two points *a* and *b*.

### 4.5 Result analysis

Experiments were conducted based on the Synapse and ACDC datasets and compared to mainstream segmentation frameworks, including V-Net (Milletari et al., 2016), DARR (Fu et al., 2020), U-Net (Ronneberger et al., 2015), Att UNet (Oktay et al., 2018), ViT (Dosovitskiy et al., 2020), and TransUNet (Chen et al., 2021). Among them, R50 indicates that the encoder of the network is composed of ResNet50 (He et al., 2016).

#### 4.5.1 Training loss

**Figure 6:**
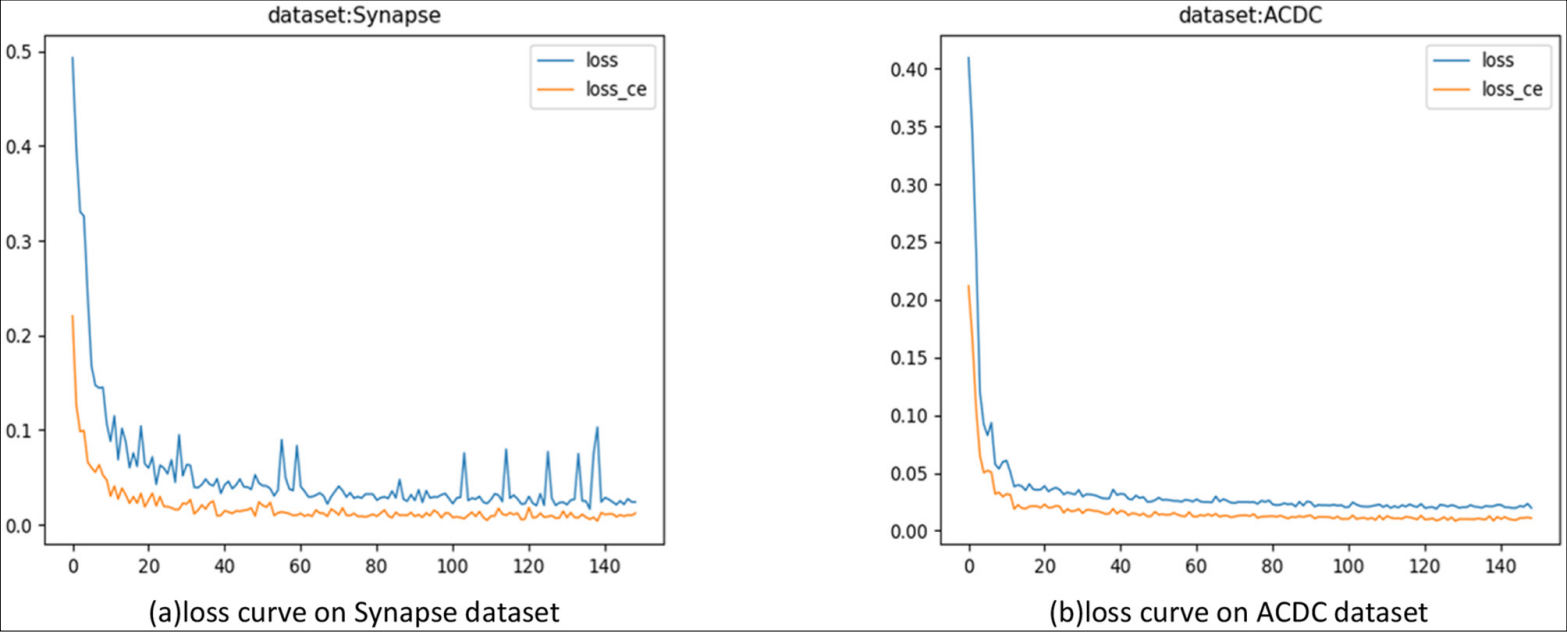
Model training loss curves. **(A)** Loss curves on the Synapse dataset; **(B)** Loss Curve on the ACDC Dataset.

In the experiments, the model was trained for 150 epochs. In each epoch, the difference between the model iteration results and the true values was calculated to guide the next training cycle toward the correct direction. As the number of iterations increased, the loss of the model gradually decreased and tended to stabilize, as shown in Figure 6.

#### 4.5.2 Synapse multi-organ segmentation dataset

The segmentation results based on the Synapse dataset are shown in Table 1, indicating that the proposed IAP-TransUNet achieved the best segmentation performance, with the DSC reaching 78.85% and the HD reduced to 28.77%. Compared to TransUNet, IAP-TransUNet increased the DSC by 1.37% and reduced the Hausdorff distance by 2.92%.

**Table 1:** Segmentation results on the Synapse dataset.

**Network**	**DSC↑**	**HD↓**	**Aorta**	**Gallbladder**	**Left kidney**	**Right kidney**	**Liver**	**Pancreas**	**Spleen**	**Stomach**
V-Net	68.81	—	75.34	51.87	77.10	80.75	87.84	40.05	80.56	56.98
R50 Att-UNet	75.57	36.97	55.92	63.91	79.20	72.71	93.56	49.37	87.19	74.95
R50 U-Net	74.68	36.87	87.74	63.66	80.60	78.19	93.74	56.90	85.87	74.16
R50 ViT	71.29	32.87	73.73	55.13	75.80	72.20	91.51	45.99	81.99	73.95
DARR	69.77	—	74.74	53.77	72.31	73.24	94.08	54.18	**89.90**	45.96
U-Net	76.85	39.70	89.07	**69.72**	77.77	68.60	93.43	53.98	86.67	75.58
TransUNet	77.48	31.69	87.23	63.13	81.87	77.02	94.08	55.86	85.08	75.62
Att-UNet	77.77	36.02	**89.55**	68.88	77.98	71.11	93.57	**58.04**	87.30	75.75
**IAP-TransUNet**	**78.85**	**28.77**	88.36	66.09	**83.45**	**80.87**	**94.19**	56.51	85.47	**75.82**

Boldface indicate the best performance.

**Figure 7:**
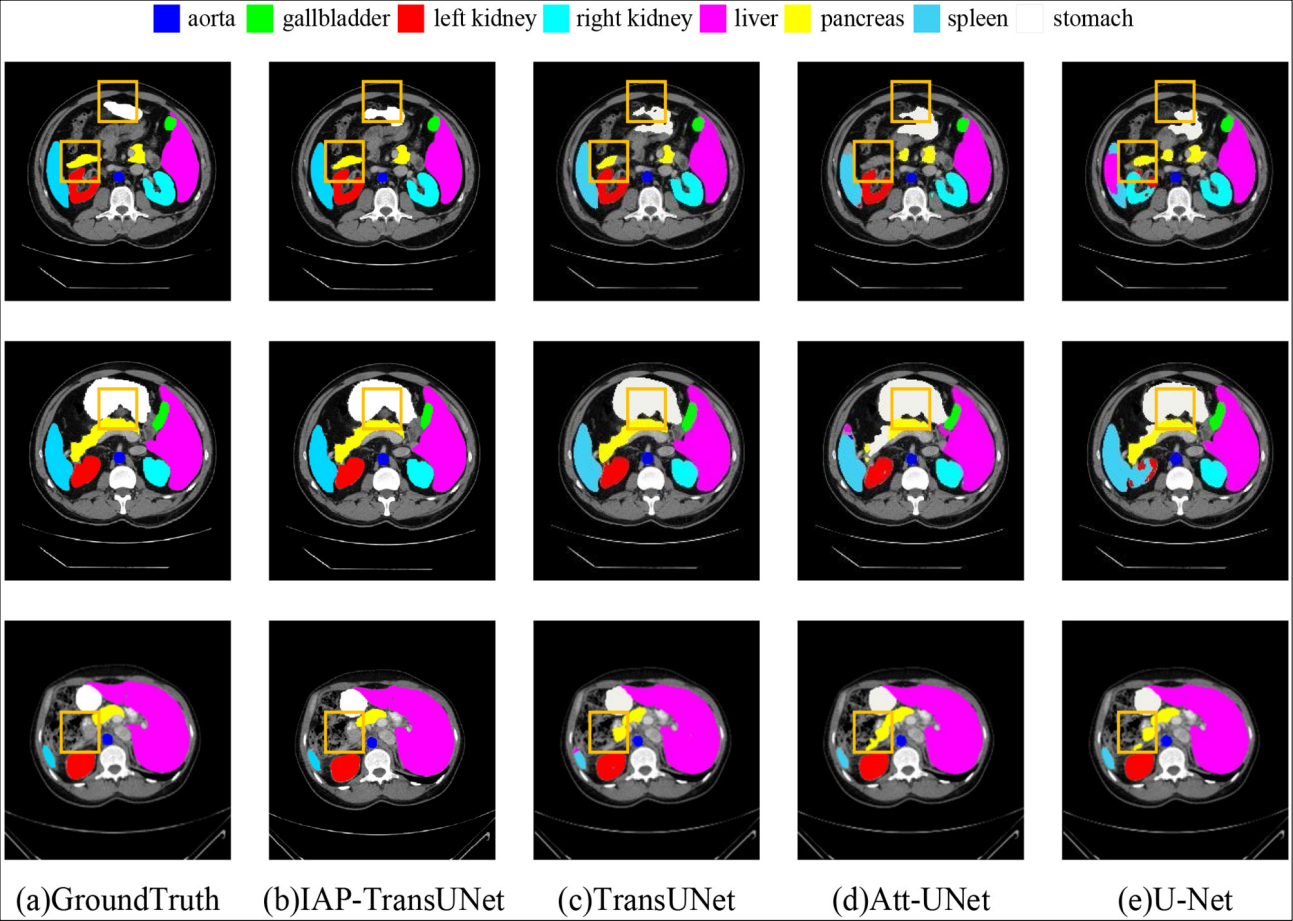
Segmentation results of the different networks on the Synapse multi-organ segmentation dataset. **(a)** Ground Truth, **(b)** IAP-TransUNet, **(c)** TransUNet, **(d)** Att-UNet, **(e)** U-Net.

The segmentation results on the Synapse dataset are shown in Figure 7, where Figures 7D, E correspond to Att-UNet and U-Net. Both suffered from insufficient or excessive segmentation of organs. From the second line, it can be seen that the spleen was insufficiently segmented by Att-UNet and excessively segmented by U-Net. TransUNet is a simple combination of a transformer and CNN, and the result is shown in Figure 7C. It only considers the fusion of local and global contextual information but fails to adequately capture detailed local features. Therefore, there are issues such as missing organs or excessive labels. From the first row and the second row, TransUNet mislabeled the pancreas, while the stomach was over-labeled. The result of the proposed IAP-TransUNet model, shown in Figure 7B, was closer to the ground truth. The experimental results showed that IAP-TransUNet paid more attention to the dependency of local contextual information, achieving better edge prediction. By integrating the ECA mechanism with the CNN and connecting to the transformer, local information with remote dependencies and global contextual information were fused together, thereby improving the feature extraction capability of the encoder. Through the multi-scale context information obtained from the CBAM_ASPP module and the efficient computation of DSC, followed by up-sampling restoration and skip connections of the U-shaped structure, a more accurate segmentation result was achieved.

#### 4.5.3 Automated cardiac diagnosis challenge dataset

The segmentation results based on the ACDC dataset are shown in Table 2. The results reflected that IAP-TransUNet showed good segmentation performance, with a Dice coefficient of 90.46%. Compared to the baseline model TransUNet, IAP-TransUNet improved the segmentation accuracy of the left ventricle, myocardium, and right ventricle by 0.14%, 1.89%, and 0.23%, respectively, outperforming mainstream models. The experimental results showed that the proposed IAP-TransUNet has good generalization ability and robustness.

**Table 2:** Segmentation accuracy of the different networks on the ACDC dataset.

**Network**	**DSC↑**	**Left ventricular**	**Myocardium**	**Right ventricular**
R50 U-Net	87.55	94.92	80.63	87.1
R50 Att-UNet	86.75	93.47	79.20	87.58
ViT-CUP	81.45	92.18	70.71	81.46
R50 ViT	87.57	94.75	81.88	86.07
TransUNet	89.71	95.73	84.53	88.86
**IAP-TransUNet**	**90.46**	**95.87**	**86.42**	**89.09**

Boldface indicate the best performance.

**Figure 8:**
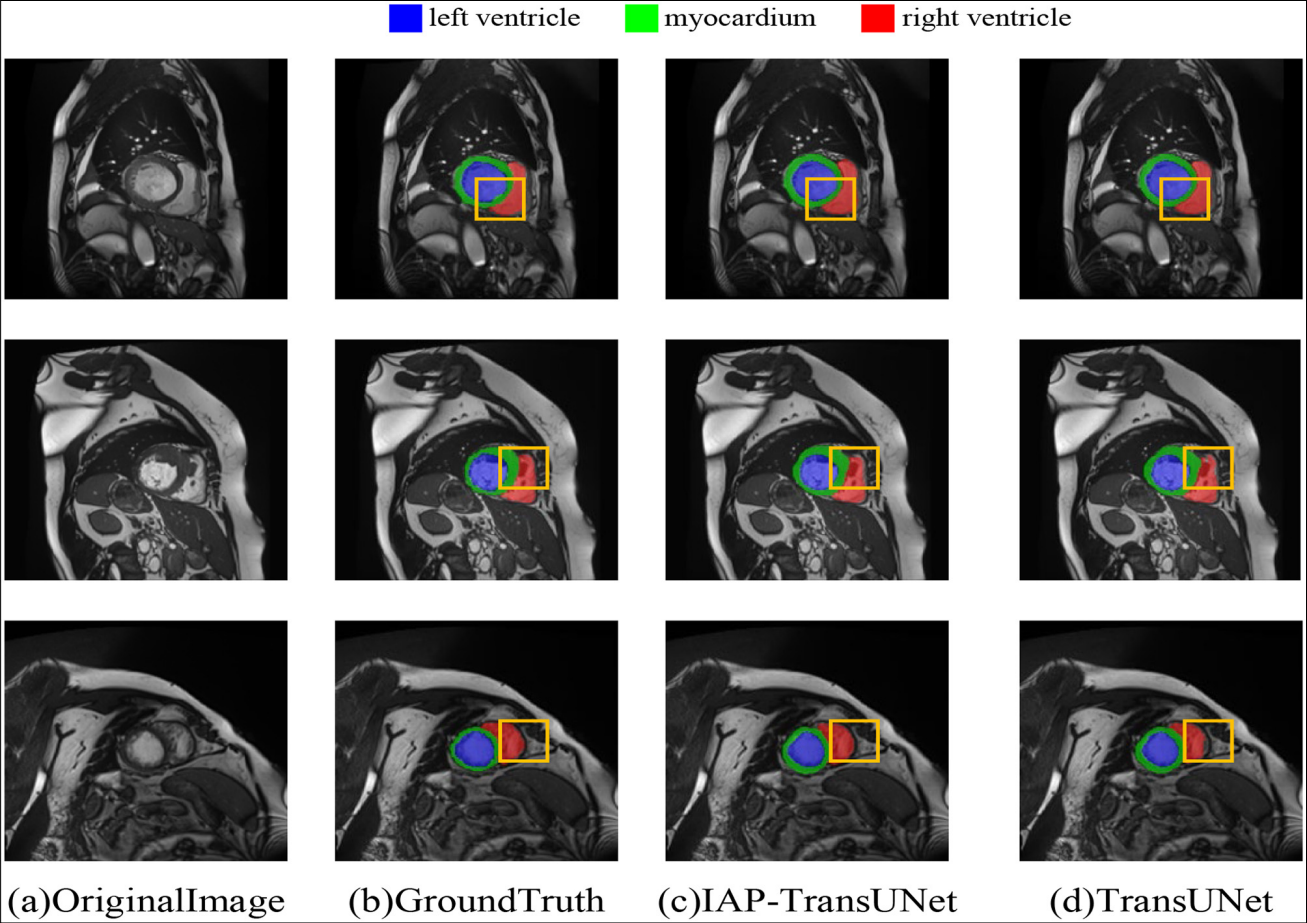
Segmentation results of the different networks on the ACDC dataset. **(a)** Original Image, **(b)** Ground Truth, **(c)** IAP-TransUNet, **(d)** TransUNet.

The segmentation results based on the ACDC dataset are shown in Figure 8, where Figure 8D displays the segmentation result of TransUNet. The first row shows excessive segmentation of the left ventricle, while the second and third rows reflect insufficient segmentation of the right ventricle. Figure 8C shows the results of the proposed IAP-TransUNet model, which performed slightly better than TransUNet in left ventricular segmentation and significantly better than TransUNet in right ventricular segmentation. The experimental results showed that the proposed IAP-TransUNet model achieves better segmentation performance compared to the baseline model TransUNet, further verifying the effectiveness of the improved model.

#### 4.5.4 Lightweight

The comparison of the number of parameters and efficiency of the different networks is shown in Table 3. According to the results, when DSC was used instead of traditional convolution in the TransUNet decoder, the number of parameters was significantly reduced to approximately half of TransUNet and the inference time and GFLOPS were reduced to approximately one-third of TransUNet, although the integration of the ECA mechanism and CBAM-ASPP may increase the number of model parameters and inference time. The experimental results showed that DSC can reduce the number of parameters and improve computing efficiency, to some extent, thereby achieving a lightweight network for the IAP-TransUNet proposed in this study.

**Table 3:** Comparison of the number of parameters and efficiency of the different networks.

**Network**	**Parameter amount (M)**	**Inference time (ms)**	**GFLOPS**
U-Net	**31.13**	223	55.84
Att-UNet	36.72	230	54.82
TransUNet	105.32	246	38.52
TransUNet+DSC	39.85	**84**	**13.08**
**IAP-TransUNet**	59.25	112	17.76

All GFLOPS were calculated based on the 256 × 256 input size, and the reported inference times represent the average duration to process a single image. Boldface indicate the best performance.

A direct analysis of the trade-off between accuracy and model size revealed the core advantage of our architecture. As shown in Tables 1, 3, the standard U-Net was the most lightweight model (31.13 M parameters) but provided lower accuracy (76.85% DSC). The baseline TransUNet was the heaviest (105.32 M) with an accuracy of 77.48% DSC. Our IAP-TransUNet (59.25 M) positioned itself optimally on this spectrum. It decisively outperformed U-Net in accuracy for a moderate increase in size while simultaneously achieving higher accuracy than the much larger TransUNet. This demonstrates a significantly improved performance-to-cost ratio, establishing our model as a more efficient and effective architecture.

### 4.6 Ablation experiments

To investigate the effectiveness of various components of IAP-TransUNet, ablation experiments were conducted based on the Synapse dataset. The experiments examined the effectiveness of the structural design, the influence of the attention mechanism, the influence of the CBAM-ASPP module expansion rate, and the influence of the convolution group number.

**Figure 9:**
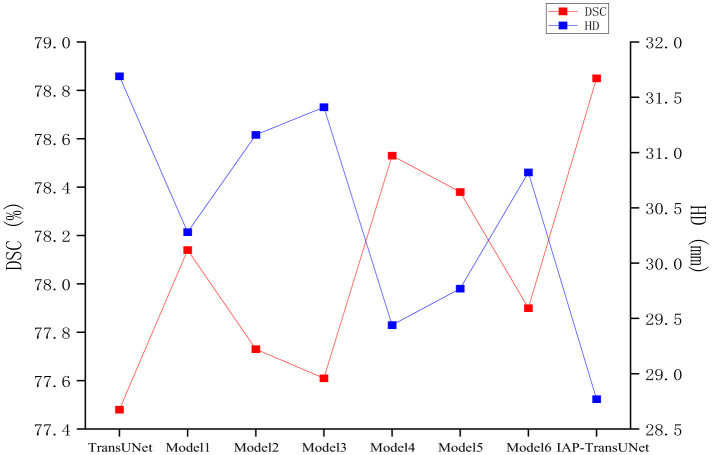
Comparison of the segmentation results of the models with different structures

(1) The effectiveness of the structural design. To verify the impact of the network architecture on segmentation performance, based on the baseline model TransUNet, six combinations of the ECA mechanism, CBAM-ASPP module, and DSC were integrated, as shown in Table 4. The segmentation results of the different combination models are shown in Figure 9. ECA injection, the CBAM-ASPP module, and DSC all improved the segmentation performance of the baseline model, achieving optimal results when fully integrated, thereby verifying the effectiveness of the structural design.
(2) Impact of the attention mechanism. To compare the impact of the ECA mechanism with other attention mechanisms, experiments were conducted by integrating the SE attention mechanism, CBAM attention mechanism, and ECA mechanism into the same network layer of TransUNet. The results are shown in Table 5. The evaluation metric DSC of the models with the SE attention mechanism, CBAM attention mechanism, and ECA mechanism increased by 0.37%, 0.53%, and 0.66%, respectively. The results indicate that the ECA mechanism can adaptively adjust the weights of channels by learning the correlations between channels, which can achieve higher segmentation accuracy.
(3) Impact of the dilation rate of the CBAM_ASPP module. To investigate the influence of different expansion rates of the CBAM-ASPP module on segmentation performance, experiments were conducted based on two sets of expansion rates: [1,2,4,8] and [3,6,9,12], and the results are shown in Table 6. Among them, the segmentation performance of the expansion rates [1,2,4,8] was better than that of the expansion rates [3,6,9,12]. Therefore, [1,2,4,8] was selected as the expansion rate for the CBAM-ASPP module.
(4) Impact of the convolution group number. To investigate the impact of convolutions with different group numbers on segmentation performance, experiments were conducted using convolutions with group numbers 1, 48, and 768, and the results are shown in Table 7. Among them, group number 1 represents ordinary convolution, and group number 768 represents deep convolution. The experimental results showed that the convolution with group number 768 achieved more accurate segmentation results, so we chose deep convolution.

**Table 4:** Ablation experiment on the structure design.

**Model**	**ECA**	**CBAM_ASPP**	**DSC**	**DSC↑**	**HD↓**
TransUNet				77.48	31.69
Model1	√			78.14	30.28
Model2		√		77.73	31.16
Model3			√	77.61	31.41
Model4	√	√		78.53	29.44
Model5	√		√	78.38	29.77
Model6		√	√	77.90	30.82
**IAP-TransUNet**	√	√	√	**78.85**	**28.77**

Boldface indicate the best performance.

**Table 5:** Ablation experiment on the attention mechanism.

**Model**	**DSC↑**	**Aorta**	**Gallbladder**	**Left kidney**	**Right kidney**	**Liver**	**Pancreas**	**Spleen**	**Stomach**
TransUNet	77.48	87.23	63.13	81.87	77.02	94.08	55.86	85.08	75.62
TransUNet+SE	77.85	87.45	63.76	81.92	78.15	94.34	56.03	85.37	75.78
TransUNet+CBAM	78.01	87.53	63.84	82.04	78.25	94.67	56.25	85.40	76.11
TransUNet+ECA	**78.14**	**87.62**	**63.97**	**82.19**	**78.36**	**94.73**	**56.47**	**85.52**	**76.28**

Boldface indicate the best performance.

**Table 6:** Ablation experiment on the dilation rate of the CBAM_ASPP module.

**Dilation rate**	**DSC↑**	**Aorta**	**Gallbladder**	**Left kidney**	**Right kidney**	**Liver**	**Pancreas**	**Spleen**	**Stomach**
[3,6,9,12]	78.73	88.25	66.01	83.35	80.80	93.94	56.45	85.38	75.67
[1,2,4,8]	**78.85**	**88.36**	**66.09**	**83.45**	**80.87**	**94.19**	**56.51**	**85.47**	**75.82**

Boldface indicate the best performance.

**Table 7:** Ablation experiment on the convolution group number.

**Group number**	**DSC↑**	**Aorta**	**Gallbladder**	**Left kidney**	**Right kidney**	**Liver**	**Pancreas**	**Spleen**	**Stomach**
1	78.57	87.82	65.18	83.02	78.49	94.23	**57.30**	**86.57**	**75.98**
48	78.30	87.98	63.59	82.97	79.56	**94.24**	55.93	86.18	75.96
768	**78.85**	**88.36**	**66.09**	**83.45**	**80.87**	94.19	56.51	85.47	75.82

Boldface indicate the best performance.

## 5 Conclusion

This study proposes a new medical image segmentation model, IAP-TransUNet, to address the limitations of convolutions in medical image segmentation networks, such as small receptive fields, insufficient information exchange between local regions, and excessive computational complexity, by integrating an attention mechanism with pyramid pooling. The innovation of this model lies in embedding an ECA mechanism in a CNN to extract more comprehensive features, introducing the CBAM-ASPP module into the bottleneck layer to obtain multi-scale contextual information and using DSC instead of traditional convolution to achieve a lightweight network for medical image segmentation. The experiment was based on the Synapse and ACDC datasets, and the results show that the proposed IAP-TransUNet achieves excellent segmentation performance and can be applied to medical images of CT, MRI, and other modalities. In terms of computational efficiency, the proposed IAP-TransUNet has fewer parameters, faster inference speed, and lower GFLOPS compared to the baseline model TransUNet.

Despite its promising results, this study has several limitations. First, while IAP-TransUNet is more lightweight than the original TransUNet, its parameter count is still higher than that of simpler architectures, such as the standard U-Net, which could be a concern for deployment on resource-limited clinical devices. Second, the segmentation of extremely small or ambiguously bordered lesions remains a challenge.

Future research will proceed in several directions. First, we will investigate advanced model compression techniques, such as network pruning and quantization, to further reduce the model's footprint without significantly compromising accuracy. Second, we plan to explore 3D extensions of IAP-TransUNet to better leverage spatial information and improve segmentation performance.

## Data Availability

The original contributions presented in the study are included in the article/supplementary material, further inquiries can be directed to the corresponding author/s.
